# Knowledge about electronic cigarettes and its perception: a community survey, Egypt

**DOI:** 10.1186/s12931-016-0365-0

**Published:** 2016-05-17

**Authors:** Omaima I. Abo-Elkheir, Eman Sobh

**Affiliations:** Community and Occupational Medicine Department, Faculty of Medicine, Al-Azhar University, Cairo, Egypt; Pulmonary Medicine Department, Faculty of Medicine, Al-Azhar University, Cairo, Egypt; Pulmonary Medicine Department, Al-Zahraa University Hospital, 11517, Al-Abbassia, Cairo, Egypt

**Keywords:** Electronic cigarettes, Knowledge, Smoking, Perception, Community survey

## Abstract

**Background:**

Electronic cigarettes are promoted as safer products than traditional cigarettes and as smoking cessation devices. Awareness and perception are key elements for the adoption of new habits. Little is known about electronic cigarettes and public opinions towards it. This study aims to identify the prevalence of knowledge about electronic cigarettes, its perception, and use among Egyptian population.

**Methods:**

An observational cross-sectional study using self-administered questionnaire in Arabic language was conducted between March and April 2015 among a sample of Egyptian population aged 15–75 years (*N* = 1239). We compared between respondent’s who know e-cigarettes and those who do not know it.

**Results:**

More than half (57.5 %) of the respondents had heard about e-cigarettes, 51.8 % were non-smokers and nobody of them reported use of e-cigarettes. There were statistically significant differences between those who know and those who do not know e-cigarettes regarding age, educational levels and history of smoking. Among those who know e-cigarettes, 41.6 % believed that e-cigarettes help smoking cessation and 31.9 % believed it is less harmful than traditional cigarettes.

**Conclusions:**

A considerable level of knowledge about e-cigarettes is found among young people aged 15–39 years. E-cigarettes were perceived as less harmful than traditional cigarettes or a smoking cessation aid. None of the respondents reported use of e-cigarettes.

## Background

The use of different forms of tobacco inflicts a huge and growing burden on public health globally. Electronic cigarettes or e-cigarettes (ECs) were invented in China in 2003 [1], these are battery-operated vaporizing devices look like a cigarette and used to deliver nicotine vapor to users [2]. A wide variety of ECs are available in the market; the main ingredients of e-cigarettes liquid other than nicotine are propylene glycol, glycerol and several flavorings [3]. E-cigarettes use and promotion appears in news stories and entertainment media particularly the internet as safer products than regular cigarettes or as medical products and smoking-cessation aids [4]. The extent to which e-cigarettes use will result in nicotine dependence and subsequent use of other tobacco products as well as the effect of these products on public health is unknown [5]. Concerns have been raised about the rising popularity and availability of e-cigarettes together with its unique nature and flavorings that may attract youth to try it [5]. A controversy was generated around e-cigarettes within the tobacco control field; some recommend using e-cigarettes as a smoking cessation aid while others debate that e-cigarettes should be banned due to lack of safety and efficacy data [6]. Furthermore, e-cigarettes are not currently approved by the Food and Drug Administration (FDA) for smoking cessation [7]. Also, the Forum of International Respiratory Societies (FIRS) recommended restriction or banning of e-cigarettes until availability of more information about their safety [8].

E-cigarettes attract smokers by providing sensation and appearance mimicking cigarettes; they are socially acceptable by enabling users to retain their smoker identity without the risk of smoke [9]. Worldwide awareness and use of e-cigarettes have dramatically increased in recent years [10] especially among youth [5]. More educated people have a higher awareness of e- cigarettes than those with lower education [4, 6].

Egypt has the highest number of tobacco users in the Arab region [11]. WHO reported 22 % of Egypt’s populations are current or former smokers, of which 43 % men and about 1 % women [12]. Also; a significant number of youth and adolescents consume tobacco products [11]. According to the Global Youth Tobacco Survey (GYTS); 13.6 % of Egyptian youth (18.1 % of boys and 8.2 % of girls) reported current use of any tobacco products [13].

Egypt has made important strides in efforts to control tobacco use and reduce its adverse health effects. Laws and regulations have been enacted to ban indoor smoking in public places and taxes on cigarette sales were imposed [14, 15], Moreover; selling tobacco products is banned by law to those less than 18 years old. Tobacco advertising, promotion, and sponsorship are also banned in Egypt by law No. 52 for 1981 [16]. However; some forms of tobacco marketing including internet advertising and product placement in movies and television programs are generally allowed [11, 17]. The recognition of tobacco use as an addiction and cause of cancer, along with concerns about the ill-effects of breathing secondhand smoke led to declining social acceptance of smoking [18], besides the advent of legal restrictions on smoking [16] make e-cigarettes an attractive alternative to traditional cigarettes in Egypt.

However, up to our knowledge, there is no data available about awareness of e-cigarettes and its use in Egypt. Therefore, assessment of the level of awareness and utilization of e-cigarettes among the population is needed. This study will be partially helpful in the analysis and evaluation of the situation in Egypt related to e-cigarettes.

### Objective

To identify the prevalence of knowledge about electronic cigarettes, its perception, and use among Egyptian population.

## Methods

### Study design

An observational cross-sectional community survey was conducted as between March and April 2015.

### Sample

This study included Egyptian population aged ≥ 15 years; recruited from different Egyptian Governorates by using convenient sample technique. Out of the 27 Egyptian governorates; 20 governorates were included in this study representing urban governorates, Upper Egypt, Lower Egypt and Frontier governorates. Among the 1294 persons who received the questionnaires; 1239 accepted to participate in this study and returned a completely filled questionnaire (response rate was 88.9 %). Data was collected by trained fourth-year medical students.

### Study tool

A questionnaire was designed in Arabic language to attain the predetermined objective of the study. The questionnaire consisted of closed-ended questions covered the following items: 1- socio-demographic characteristics as age, gender, and residence, 2-level of education, either informal, preparatory, secondary or above secondary education, 3- history of smoking (either current/or ex-smoker or nonsmoker), 4-Knowledge about e-cigarettes were assessed by asking them” have you ever heard about e-cigarettes?”, then those answered “yes” were asked about the main source (s) of their knowledge, either through reading journals and newspapers, mass media & internet or from friends, 5- Use of e-cigarettes was assessed by asking them “if you ever used or tried e-cigarettes?, 6- Perception about e-cigarettes: participants were asked the following question: Do you believe that e-cigarettes, are (less harmful, equally harmful, or more harmful) than traditional cigarettes?; and if e-cigarettes help smoking cessation.

A pilot testing was done on a small group (25 subjects) in order to check clarity and adjust the wording of the questionnaire. Results of pilot testing were not included in this study.

### Ethics, consent, and permissions

The study was approved by the ethical review committee of the faculty of medicine for girls Al-Azhar University. An informed verbal consent was obtained from all participants after they were informed about the aim of the study. Also, confidentiality of data was assured as the survey tool was anonymous, no reimbursements or prizes were offered to the participants and participation was voluntary.

### Statistical analysis

Collected data were statistically analyzed by Statistical Package for Social Science (SPSS) program version 17.0. Descriptive statistics were used to summarize characteristics of the sample. Qualitative (categorical) data were presented by percentages, while, quantitative data were presented by mean ± SD.

The studied sample was categorized into two groups based on their knowledge about e-cigarettes. The difference between the groups was assessed using Chi-square (X^2^) test for categorical data and Student t-test for quantitative data. Statistical significance was considered at *p* - value < 0.05 (with a confidence limit at 95 %). Results were presented by tables and figures.

## Results

### Characteristics of the studied sample

**Fig. 1:**
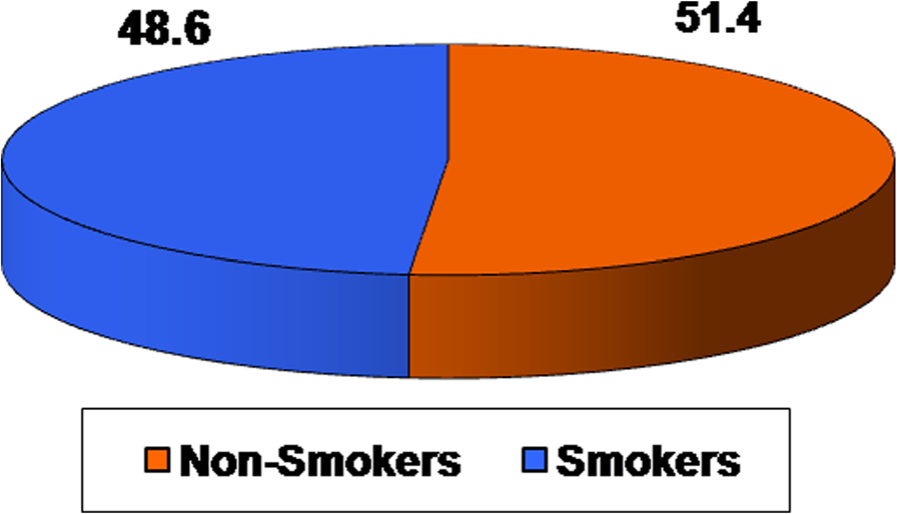
Smoking status of the sample

A sample of 1239 of Egyptian population was included in this study, their age ranged from 15–75 years (33.3 ± 13.8 years). Three-quarters of them were males and one-quarter was females. The majority of the studied sample (74.6 %) reported education level above secondary education, 9.5 % had secondary education, 6.4 had preparatory education and the remaining 9.5 % had informal education. The highest proportion of the participants (60.2 %) was from Lower Egypt governorates. Around half of the participants (48.6 %) were smokers (94.7 % males and only 5.3 % females) (Table 1 and Fig. 1).

**Table 1:** General characteristics of the studied population

Items	Total No. = 1239 (100 %)
	%
Age /years	
15–24	37.7
25–39	29.5
40–54	22.7
55+	10.1
Mean ± SD	33.3 ± 13.8
Sex	
-Male	75.4
-Female	24.6
Residence by governorate	
-Urban governorate	20.5
-Lower Egypt governorate	60.2
-Upper Egypt governorate	16.7
-Frontier governorate	2.6
Educational level	
-Informal education	9.5
-Preparatory education	6.4
-Secondary education	9.5
-Above secondary education	74.6
History of smoking	
-Non- smoker	51.4
-Smoker	48.6
Male	94.7
Female	5.3

### Awareness of E-cigarettes

**Fig. 2:**
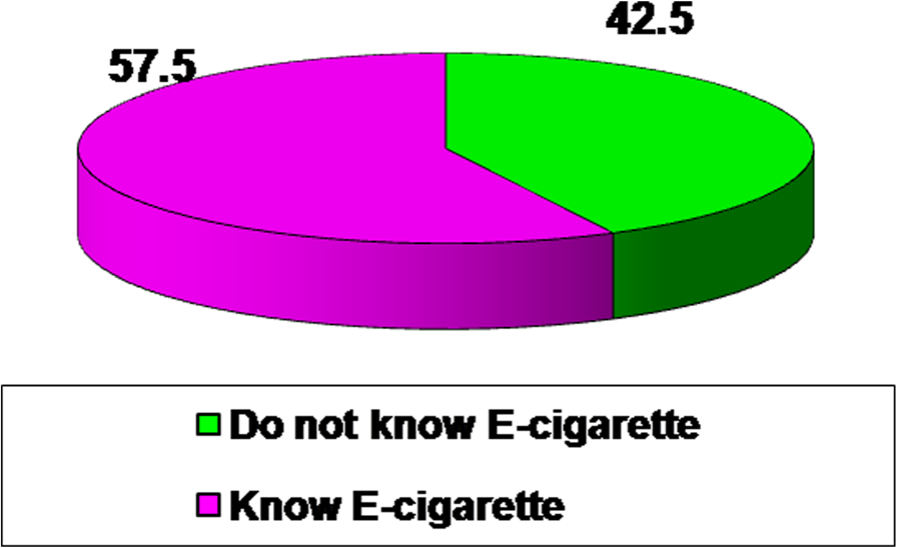
Knowledge of e-cigarette among the studied sample

More than half (57.5 %) of the participants had heard about e-cigarettes versus 42.5 % never heard about it with a statistically significant differences regarding age, educational levels and history of smoking (Fig. 2, Table 2).

**Table 2:** Comparison between the studied groups regarding general characteristics according to knowledge of E- cigarette

Group items	Know E-cigarette no. = 713 (57.5 %)	Do not Know E-cigarette no. = 526 (42.5 %)	Sig. test & *p*-value
	%	%	
Age /years			
15–24	42.3	31.2	Chi square (X^2^) = 35.2 *P* = 0.000*
25–39	31.3	27.2	
40–54	19.6	26.9	
55+	6.8	14.7	
Mean ± SD	31. 4 ± 12.6	35. 8 ± 14. 8	t - test = 31.3
			*p* = 0.000*
Sex			
-Male	73.5	78.0	Chi square (X^2^) = 3.3
-Female	26.5	22.0	*P* = 0.07
Educational level			
-Informal education	4.8	15.7	Chi square (X^2^) = 68.8
-Preparatory education	4.1	9.4	*P* = 0.000*
-Secondary education	8.2	11.3	
-Above secondary education	82.8	63.5	
History of smoking			
-non- smoker	54.3	48.5	Chi square (X^2^) = 4.1
-Smoker	45.7	51.5	*P* = 0.04*
Male	93.0	96.7	
Female	7.0	3.3	

*Significant level of *p*-value

### Use of e- cigarettes

None of the studied population reported use of e- cigarettes.

### Perception of e-cigarettes

**Fig. 3:**
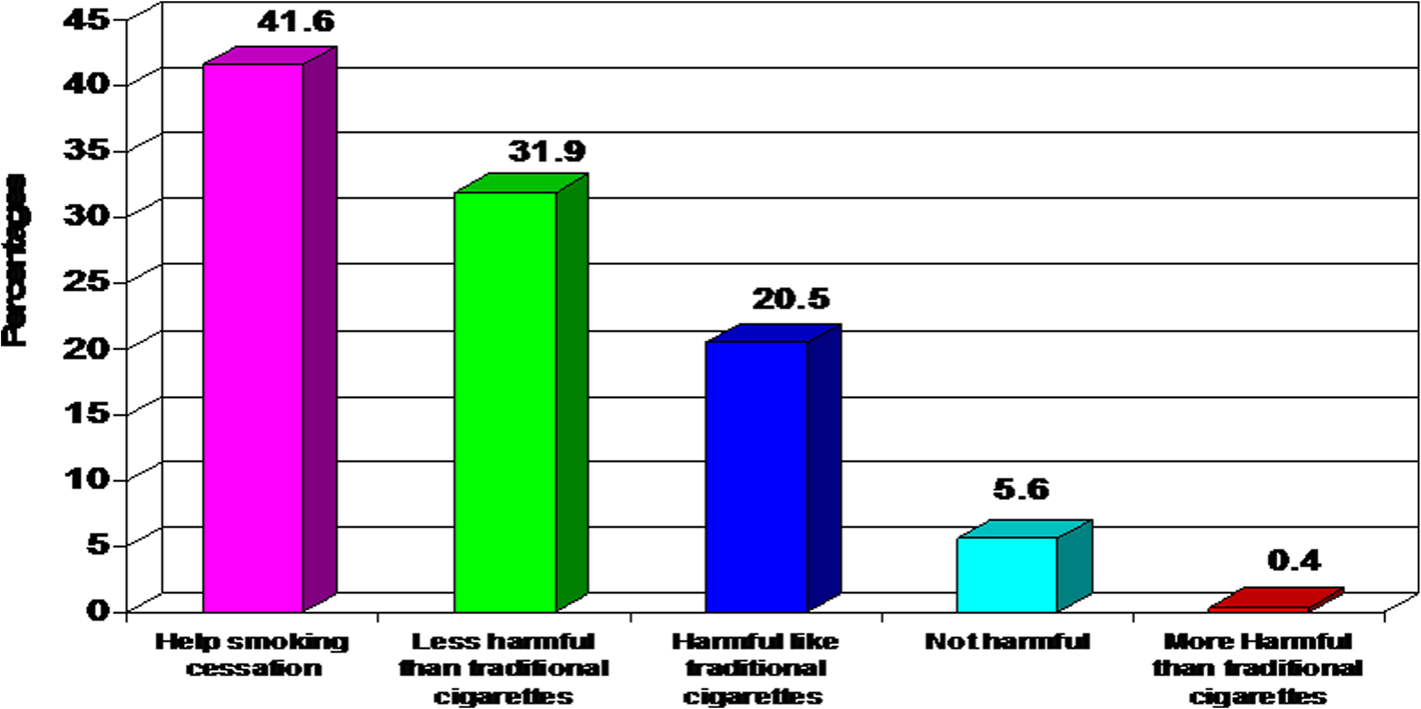
Perception of respondents about e- cigarettes among those who know it

Among respondents who know e-cigarettes, more than one-third (41.6 %) of them believe that e-cigarettes help smoking cessation, 31.9 % believe that e-cigarettes are less harmful than traditional cigarettes and 5.6 % believe it is not harmful at all. However, 20.5 % mentioned it is harmful like traditional cigarettes and only 0.4 % reported it is more harmful than traditional cigarettes (Fig. 3).

### Sources of respondents’ knowledge about e-cigarettes

**Fig. 4:**
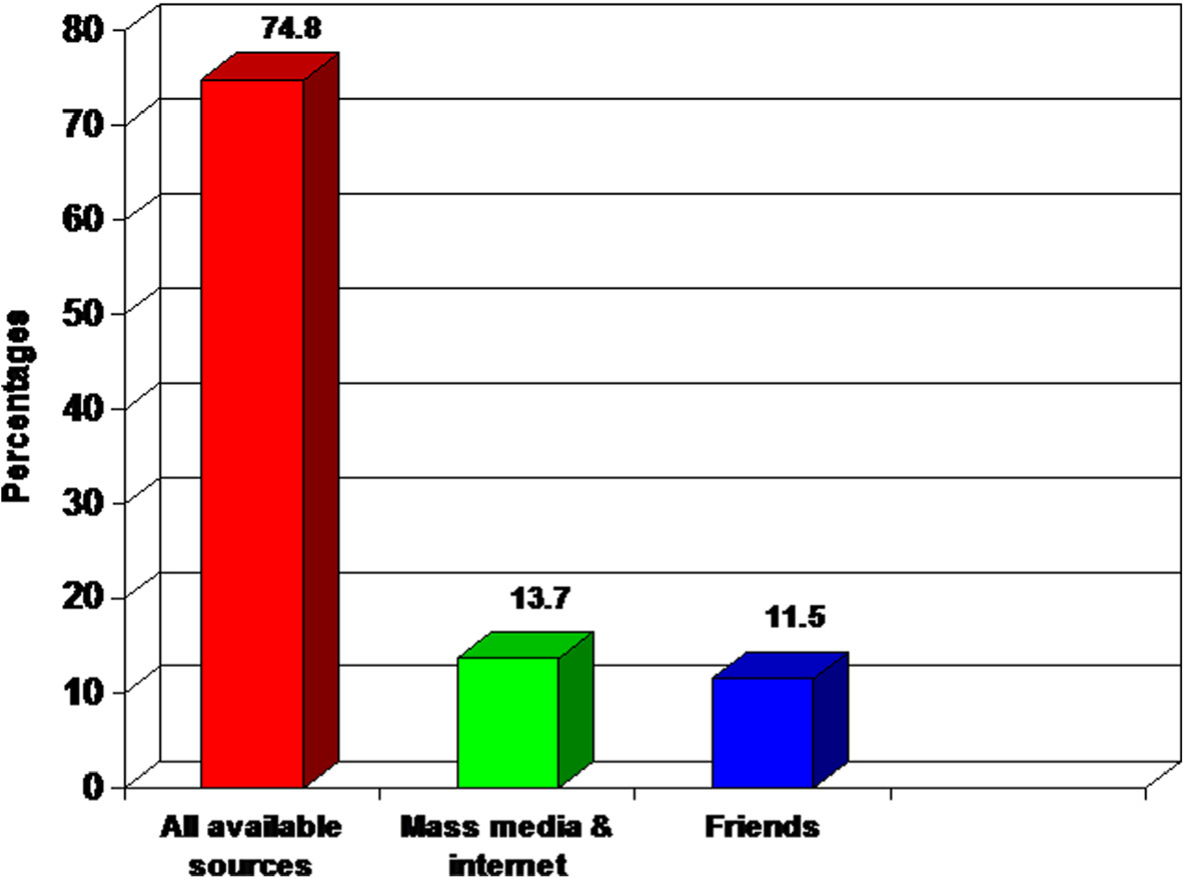
Sources of information about e-cigarettes among those who know it

The majority (74.8 %) of those who are aware of e-cigarettes gained their knowledge from multiple available sources (like friends, mass media, the internet and others), 13.7 % from mass media and the internet and 11.5 % from friends (Fig. 4).

## Discussion

There is both considerable interest and debate around the consequences of e-cigarettes use on tobacco control and public health. Data about the rate of ECs awareness and use are limited to some countries [19, 20]. However, some studies reported a significant increase in awareness and use of e-cigarettes over recent years [21, 22]. To date, national data on awareness, perception and use of ECs among the Egyptian population are unavailable, and the research that has been conducted primarily focused on traditional methods of smoking [11]. There is a need to identify what the public knows and believes about e-cigarettes, as well as who uses EC. Accordingly, the current survey was conducted as an exploratory study.

Findings of this study revealed that 57.5 % of respondents had heard about e-cigarettes compared to 42.5 % didn’t hear about it at all. Knowledge rate was higher among males (73.5 %) than females (26.5 %) (Fig. 2 and Table 2). Various studies reported high awareness rates of e-cigarettes [6, 23–25] and men were more likely to be aware of ECs than women [26, 27]. In our study; younger respondents were more aware of ECs than older respondents; the highest percentage of awareness (42.3 %) was among group aged 15–24 years while the lowest percentage (6.8 %) was among those aged ≥ 55 years (Table 2). These findings have been reported elsewhere [4, 23, 26–28]. This may be attributed to the curiosity of youth towards new devices and their higher use of the internet and social media where e-cigarettes are marketed and promoted. E-cigarettes awareness among youth and their desire for experimentation might have an impact on the initiation of tobacco use and nicotine addiction [29].

The rate of knowledge about e-cigarettes in the current study was significantly related to the educational level, where 82.8 % of those who know e-cigarettes have a level of education above secondary compared to 63.5 % among those who do not know e-cigarettes. Also, 15.7 % of respondents who never heard about e-cigarettes didn’t receive formal education, compared to 4.8 % among those who ever heard about it with a significant *p*-value (*p* = 0.000) (Table 2). This may be due to the fact that educated people have access to information sources and their high and easy use of the communication technology [30]. These results were supported by Zhu et al. [6], who reported a high rate of e-cigarettes awareness across gender, age and education level.

Additionally, our study demonstrates that knowledge about e-cigarettes was not related to smoking status; as 54.3 % of those who had heard about e-cigarettes were non-smokers versus 45.7 % were smokers (Table 2). Studies in other countries found contradicting patterns of awareness [4, 6, 23, 24, 31, 32] as they reported smokers were more likely to have heard of ECs than nonsmokers and current smokers were more aware than former smokers. This difference may be due to the predominance of nonsmokers in our study (51.4 % nonsmokers versus 48.6 % smokers) and the variation in age and education level.

Among our surveyed population, none of them reported use of e-cigarettes. In the same context, other studies reported high awareness level and low e-cigarettes use [23, 24]. Also, Zhu et al., reported a high proportion (75.4 %) heard about e-cigarettes, of them 8.08 % had tried e-cigarettes and 1.44 % were current users [6].

The consequences of increasing widespread awareness of e-cigarettes among the general population are still unclear. Also, whether the population perceptions about the reduced harm of e-cigarettes could promote behaviors of continuing smoking or quitting smoking is still uncertain [4].

Regarding perception of our respondents about e-cigarettes; more than one-third of those who know e-cigarettes (41.6 %) believe that use of e-cigarettes help smoking cessation. Meanwhile, 31.9 % believe that ECs are less harmful than traditional cigarettes and 5.6 % reported ECs are not harmful at all (Fig. 3). The same results were reported in various studies [4, 6, 24, 25, 29, 31].

It is not surprising that most people think e-cigarettes are safer than traditional cigarettes due to the deceiving marketing of competing advantages of e-cigarettes over traditional cigarettes.

The main source of knowledge about e-cigarettes in this survey was not specific as 74.8 % of them heard about e-cigarettes from multiple available sources, 13.7 % from mass media and the internet and 11.5 % from friends (Fig. 4). The most common sources reported in previous studies [33, 34] were the internet, friends or personal contacts, and advertisements. A study conducted by Martínez-Sánchez et al. [25] reported that most participants had learned about e-cigarettes through traditional media. Furthermore, Zhu et al., [6] found that older people and lower education groups were more likely to hear about e-cigarettes from television while the internet was the main source of knowledge in the younger people and higher education groups. Meanwhile, other studies found that information on e-cigarettes spread widely through social media [15, 35].

This study considered as an exploration of the situation of ECs awareness and use among Egyptian population. In spite of zero levels of e-cigarettes usage among the studied sample; it may present a significant public health hazard in future. Given that individuals’ perceptions affect their behavior [36], the perception that e-cigarettes used for smoking cessation or less harmful than traditional cigarettes found in this study may promote progression to e-cigarettes use among non-tobacco users, long-term dual use among current smokers, and relapse of smoking among former smokers.

## Conclusion

Our findings revealed that more than half of the studied population (57.5 %) had heard about e-cigarettes though nobody reported use of e-cigarettes. In addition, 41.6 % believed that e-cigarettes help smoking cessation; 31.9 % considered ECs less harmful than traditional cigarettes. The main source of knowledge about ECs was not specific where 74.8 % of them heard about e-cigarettes from multiple available sources. Accordingly there is a strong need to pass laws for the regulation of e-cigarettes advertising and sale in Egypt until availability of safety data. As well as the implementation of effective tobacco control strategies focused on preventing initiation and use of tobacco products. Moreover, development of educational messages reducing the appeal of ECs use and perception. Surveillance of emerging use patterns of e-cigarettes among Egyptian population is needed.

## Abbreviations

ECs: electronic cigarettes
ENDS: electronic nicotine delivery devices
FDA: Food and Drug Administration
GYTS: Global Youth Tobacco Survey
